# A rare case of melanoma erysipeloides

**DOI:** 10.1002/ccr3.6315

**Published:** 2022-09-14

**Authors:** Mariem Rekik, Chaima Kouki, Nadine kammoun, khadija Sellami, Mariem Triki, Sonia Boudaya, Emna Bahloul, Mariem Amouri, Tahya Boudawara, Hamida Turki

**Affiliations:** ^1^ Department of Dermatology Hospital of Hedi chaker Sfax Tunisia; ^2^ Department of Anatomopathology Hospital of Habib bourguiba Sfax Tunisia

**Keywords:** carcinoma erysipeloides, melanoma

## Abstract

Malignant melanoma presenting as an inflammatory skin metastasis has been described but is an exceedingly rare phenomenon. We report a case of inflammatory metastasis of cutaneous melanoma (CM).

## Graphical abstract

Carcinoma erysipeloides remains rare form of cutaneous metastasis that presents as an erythematous, well‐demarcated patch or plaque that resembles erysipelas. It is most frequently associated with carcinoma of the breast, but has been reported with other primary tumors, including lung and colon, accounting for 4% of skin metastases. In this report, we describe a case of inflammatory metastasis of cutaneous melanoma (CM).

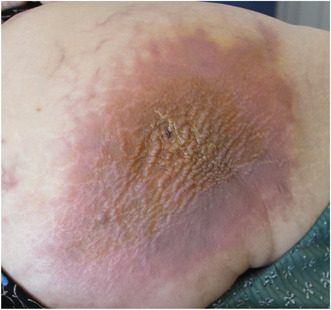

A 76‐year‐old woman was admitted for 1 month evolving painful indurate plaque on the right buttock without fever. On physical examination, the patient had a 10 cm plaque with multiple erythematous nodules (Figure 1). CT scan showed multiple metastatic mass in the lung and stomach with nodular tissue thickening of the buttock. Biopsy showed subcutaneous infiltration by atypical cells with an eosinophilic cytoplasm and hyperchromatic nuclei.

**FIGURE 1:**
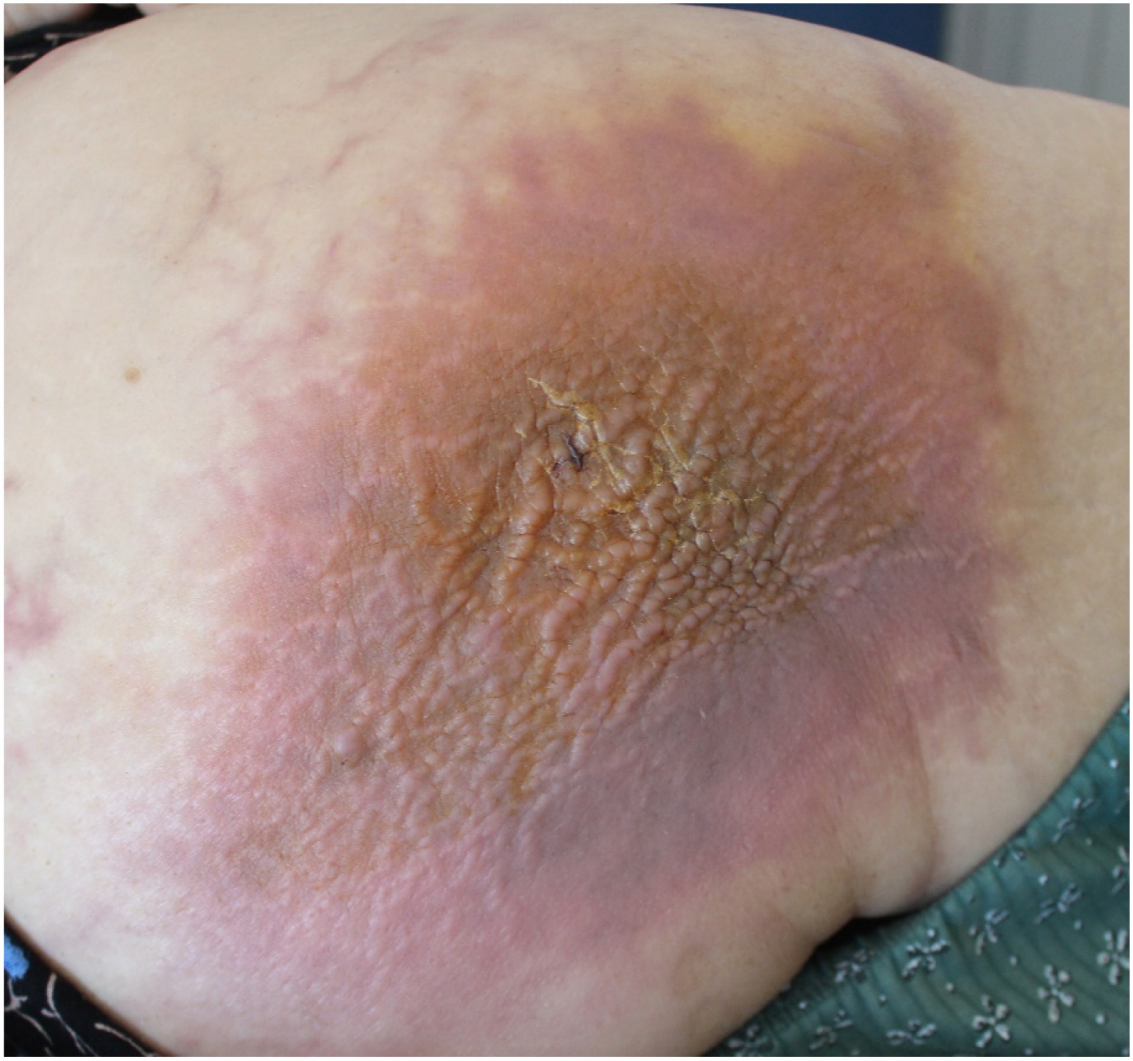
Erythematous and infiltrated plaque of the right buttock measuring 10 cm

The tumor cells were positive for pS100. (Figure 2). The patient reported a history of surgical excision of a CM located on the right breast 2 years ago.

**FIGURE 2:**
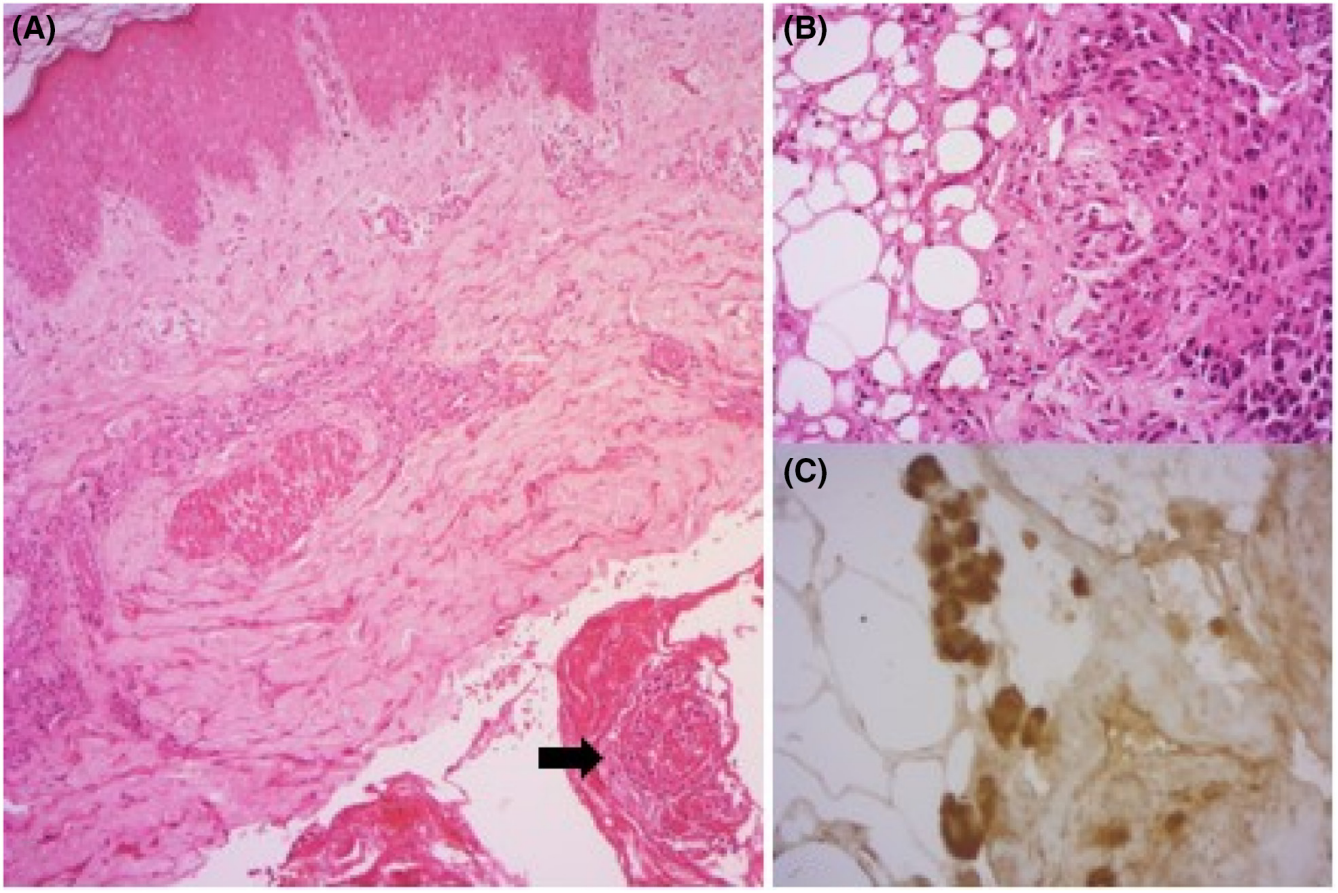
Metastatic melanoma to the right buttock. (A) Sheets of atypical cells in the hypodermis (arrow) (HEx50), (B) At high magnification, the tumor cells, invading the fat connective tissue, are round with eosinophilic cytoplasm and hyperchromatic nuclei (HEx200), (C) The tumor cells are positive for pS100 (x400).

Diagnosis of melanoma erysipeloides (ME) was made. Because of the advanced tumor stage and the limited treatment options in our country, palliative attitude was recommended.

Malignant melanoma presenting as an inflammatory skin metastasis is an exceedingly rare phenomenon.^1^ To the best of our knowledge, there are only 11 reported cases of ME.^2^

In our patient, clinical aspect of the lesion, absence of fever, and negative biological findings were suggestive of ME. Differential diagnosis between erysipelas and ME may be difficult. Histology remains the main key for diagnosis.^2^

While cases of ME have been located around the primary tumor, in the skin overlying regional lymph node metastases,^2^ our patient's location is quite unusual.

## AUTHOR CONTRIBUTIONS

Dr kouki chaima and rekik mariem wrote the manuscript and are the guarantors of the content of the manuscript, including the data and analysis. Dr kammoun nadine involved in analysis and interpretation of data and revised it critically. Dr Sellami khadija and Bahloul emna contributed to interpretation of data and revision of the manuscript. Dr boudaya sonia and amouri mariem contributed to data collection and approved the final manuscript. Dr triki mariem provided the antomopathological figures. Dr Hamida Tuki and Dr boudawara tahya contributed to the final approval of the version of the manuscript to be submitted.

## CONFLICT OF INTEREST

None.

## ETHICAL APPROVAL

Approved.

## CONSENT

Approved by all authors. Written informed consent was also obtained from the patient to publish this report in accordance with the “journal’s patient consent policy”.

## Data Availability

not available
